# Left Subclavian and Innominate Vein Balloon Venoplasty Followed by Permanent Pacemaker Implantation: A Case Report

**DOI:** 10.19102/icrm.2019.100704

**Published:** 2019-07-15

**Authors:** Balijepalli G. Sudhakar

**Affiliations:** ^1^Department of Cardiology, KIMS Hospital, Secunderabad, India

**Keywords:** Balloon venoplasty, central venous catheters, pacing leads, upper-extremity dep vein thrombosis, venous stenosis

## Abstract

Upper-extremity venous obstruction is not an uncommon problem encountered by electrophysiologists. The placement of any catheter including pacemaker leads can cause stenosis or total obstruction. Affected patients often require balloon venoplasty to facilitate lead implantation. If the vein is unresponsive to venoplasty, stenting of the vein should be contemplated. We report a case of permanent pacemaker implantation after balloon venoplasty of the left subclavian vein and innominate vein following total occlusion in a patient with symptomatic complete heart block. There are many case reports to date in which balloon venoplasty of the subclavian vein has been performed before upgrading a single-chamber pacemaker to a DDD-mode pacemaker, cardiac resynchronization therapy device, or implantable cardioverter-defibrillator because of chronic venous occlusion secondary to a preexisting pacing lead. Balloon venoplasty to increase the diameter of a target vein or to overcome stenosis may be a technique that implanting electrophysiologists could adopt in order to achieve success in patients with more challenging anatomies.

## Introduction

Most critically ill patients will require a central line placement at some point. Any catheter placement including pacemaker leads can irritate the vein endothelium and cause stenosis or total obstruction. Upper-extremity venous stenosis and obstruction occurs overwhelmingly due to indwelling devices such as central venous catheters (CVCs), hemodialysis catheters, pacemaker or defibrillator leads, and tunneled central venous access lines.^1–3^ These patients often require subclavian balloon venoplasty for the correction of access dysfunction.^4^ If the vein is unresponsive to venoplasty, vein stenting should be explored as an alternative option. Catheter usage leading to upper-extremity deep vein thrombosis (UEDVT) is the most common etiology of said condition, making up 93% of all UEDVT cases in one retrospective analysis of 373 patients.^1^ The presence of a CVC increases the risk of developing UEDVT by up to 14-fold.^5^ The present case details an instance of permanent pacemaker implantation after balloon venoplasty of the left subclavian vein and innominate vein following the observance of total occlusion in a patient who underwent therapy for right breast malignancy. There are many case reports where balloon venoplasty of the subclavian vein was performed before upgrading a single-chamber pacemaker to a DDD-mode pacemaker, cardiac resynchronization therapy (CRT) device, or implantable cardioverter-defibrillator (ICD) because of chronic venous occlusion secondary to preexisting pacing lead. However, in most of these cases, venous stenosis or thrombosis occurred in a vein with preexisting leads. Balloon venoplasty to enhance the diameter of a target vein or to overcome stenosis may be a technique that implanting electrophysiologists might consider adopting in order to achieve success in more challenging anatomies.^6^

## Case presentation

**Figure 1:**
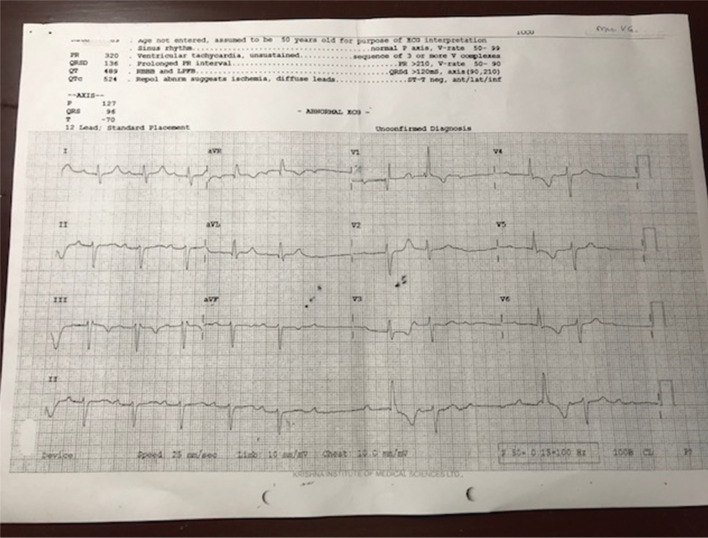
ECG showing a prolonged P–R interval, right bundle branch block, left-axis deviation, and intermittent CHB.

**Figure 2:**
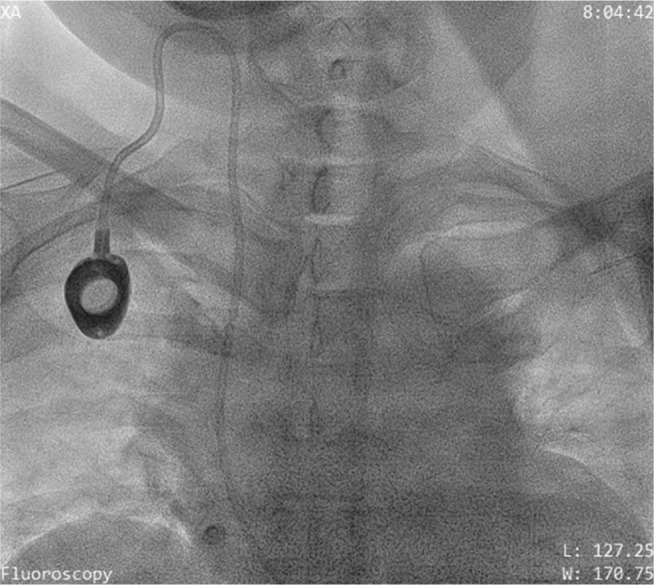
Central venous port implanted through the right internal jugular vein.

A 67-year-old female was brought to the emergency room for three episodes of unprovoked syncopal attack. Electrocardiogram (ECG) monitoring showed long pauses with atrial activity but no ventricular activity and additionally revealed trifascicular block with intermittent complete heart block (CHB) **(Figure 1)**. She reported a history of right breast malignancy, hypothyroidism, diabetes, and hypertension. She had been subjected to modified radical mastectomy, radiotherapy, and intravenous chemotherapy previously in 2009. Chemotherapy was delivered through a venous access port implanted through the right internal jugular vein **(Figure 2)**. Subsequently, she had experienced a recurrence with metastasis to the bone and lungs. At the time of the current presentation, she was taking 50 mg of atenolol in tablet form (Aten; Zydus Cadila, Ahmedabad, India), 125 mg of palbociclib in tablet form (Palbace; Pfizer, New York, NY, USA), and 2.5 mg of letrozole in tablet form (Healing Pharma, Mumbai, India) daily. A 24-hour Holter recording was obtained after cessation of the atenolol for 72 hours, which revealed long pauses with atrial activity and no ventricular activity. Considering the patient’s symptoms and long cardiac pauses, a permanent pacemaker was implanted after discussing the role of the pacemaker with both the patient and her family.

### Procedure

**Figure 3:**
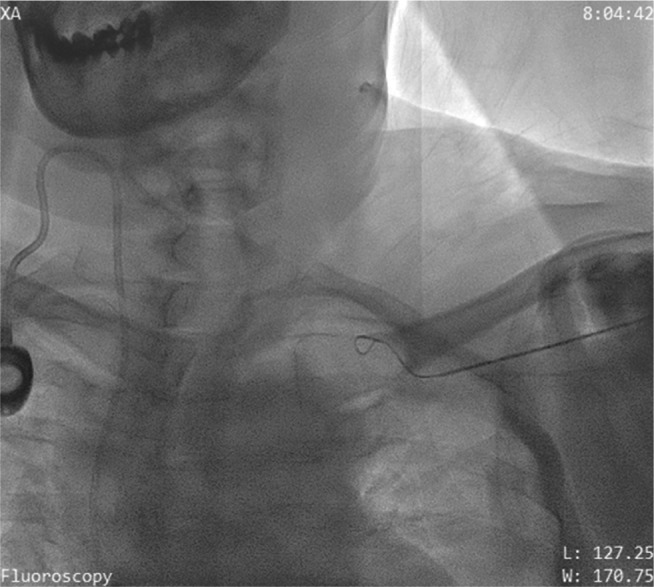
A 0.032-in guidewire (Runthrough^®^ hydrophilic 0.014-in, 180-cm-long PTCA guidewire; Terumo Medical Corp., Somerset, NJ, USA) being negotiated through the occluded left axillary vein.

**Figure 4:**
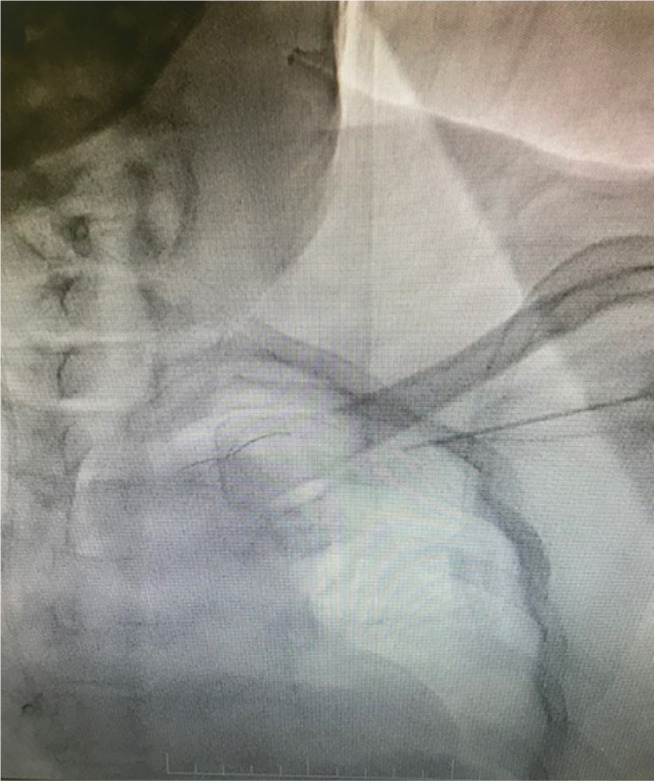
A PTCA guidewire being negotiated across the distal subclavian vein.

**Figure 5:**
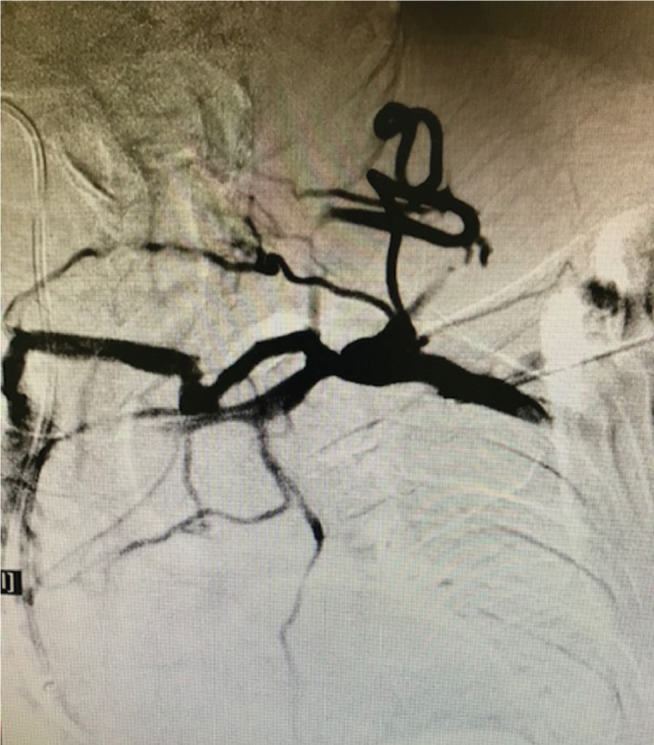
Venogram with Seldinger needle showing distal total occlusion of the left subclavian vein with extensive collaterals.

**Figure 6:**
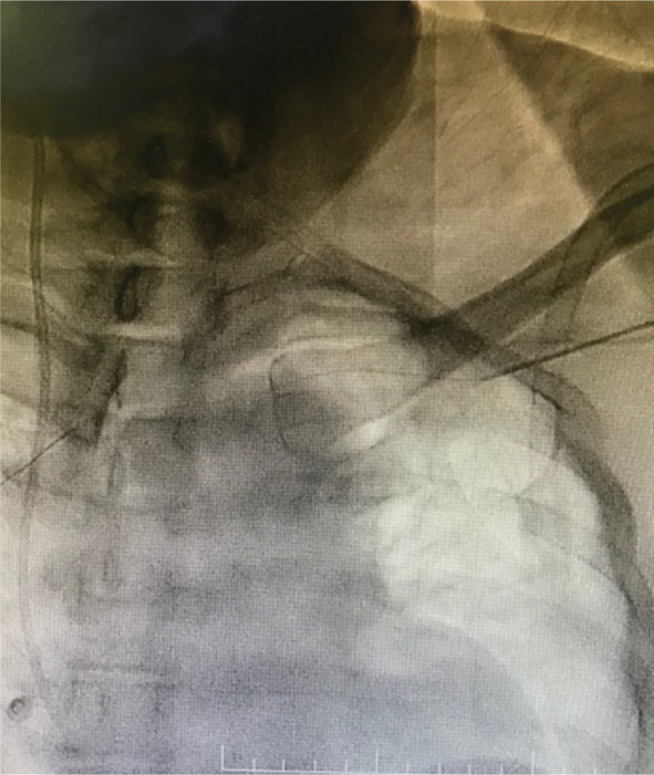
A PTCA wire located across the left innominate vein.

**Figure 7:**
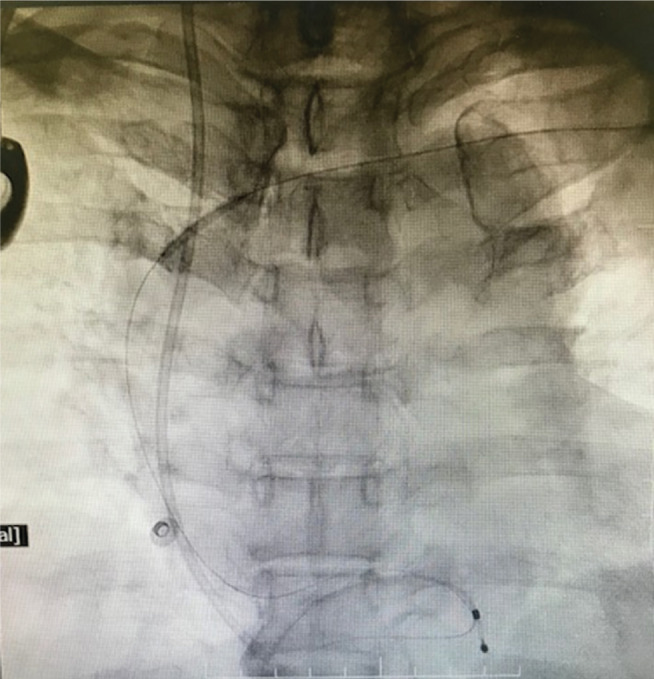
Venoplasty with a 2-mm × 10-mm coronary PTCA balloon.

**Figure 8:**
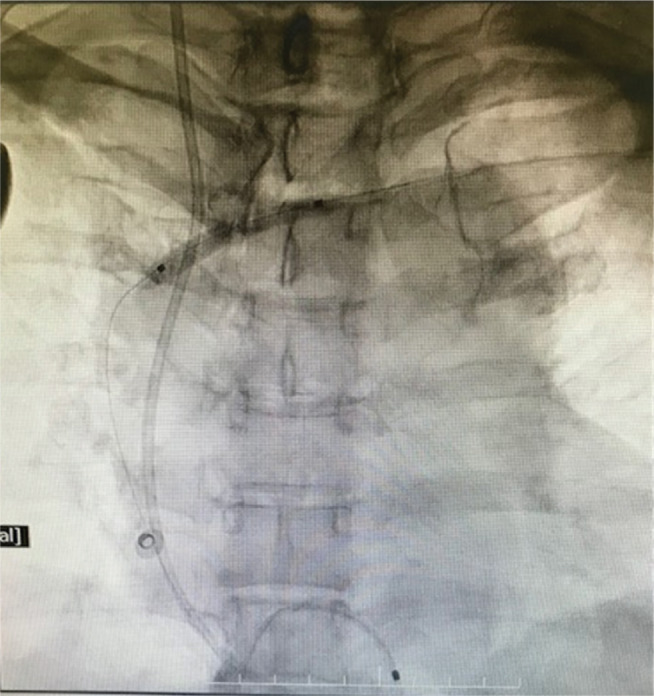
Venoplasty with a 4-mm × 20-mm peripheral balloon.

**Figure 9:**
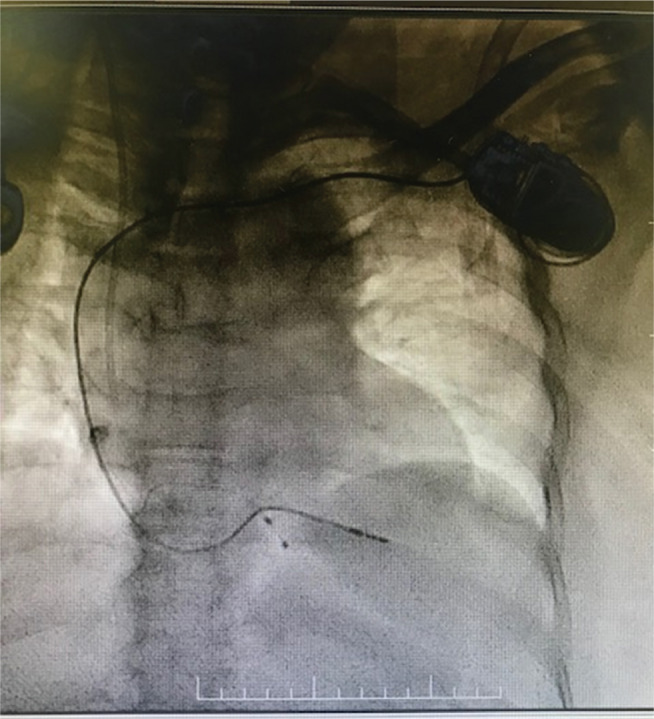
After implantation of the pacing lead and pulse generator.

Under local anesthesia, a temporary pacing lead was inserted via the right femoral vein and placed at the right ventricular apex. Additionally after local anesthesia, the left axillary vein was punctured using an 18-gauge Seldinger needle. When a 0.032-in guidewire inserted through the needle met with resistance **(Figures 3 and 4)**, it was withdrawn and contrast was injected through the needle **(Figure 5)**. Venography revealed distal total occlusion of the left subclavian vein and extensive venous collaterals. At this point, a 0.014-in percutaneous transluminal coronary angioplasty (PTCA) guidewire was negotiated across the subclavian vein and innominate vein into the inferior vena cava under fluoroscopy **(Figure 6)**. A 6-French sheath was then threaded over the PTCA wire into the axillary vein and the distal subclavian and left innominate vein were dilated serially with a 2-mm × 10-mm coronary PTCA balloon followed by a 4-mm × 20-mm peripheral balloon **(Figures 7 and 8)**. Subsequently, an active fixation (screw-in) pacing lead was implanted into the right ventricular apex **(Figure 9)**.

## Discussion

The present report details an interesting case of a patient with total occlusion of the left subclavian and innominate veins in need of permanent pacemaker support for third-degree atrioventricular block. At the time of presentation, this 67-year-old female was undergoing palliative oral chemotherapy for bone and lung metastasis from a previous right breast malignancy. The patient denies having had a prior CVC in the left side. The left subclavian and innominate veins could have therefore been thrombosed due to underlying malignancy. Venous stenoses or occlusions are often associated with the presence of multiple pacemaker/ICD leads; a history of venous thrombosis; use of temporary pacing lead before permanent pacemaker implantation; use of hormone therapy; and temporary venous access for hemodialysis, chemotherapy, and parenteral nutrition. It has also been described in patients with malignancy and genetic coagulation abnormalities. These stenoses are often fibrotic in nature, leading to a reduction in vessel lumen diameter. Most of the cases reported to date discuss venoplasty of the subclavian and innominate vein performed for upgrading existing devices.^7–9^ There are also a good number of case reports where venoplasty of the coronary sinus vein tributaries was completed to facilitate the implantation of a left ventricular lead.^10^ This is an expanding population of patients with existing pacemaker leads who require upgradation to either a DDD-mode pacemaker, ICD, or CRT pacemaker or defibrillator device. While some degree of venous obstruction has been reported in almost 15% of patients prior to device implantation, this percentage can increase up to 50% after transvenous device implantation. One major complication is perforation or rupture of the vein during venoplasty or passage of the lead following balloon venoplasty. Therefore, the utmost caution must be exercised during balloon dilatation and while passing the lead across innominate vein and the superior vena cava junction. Venoplasty and implantation of pacing leads is feasible and safe and enhances the rate of success in challenging cases. It is important for device implanters to be familiar with interventional equipment and techniques such as balloon venoplasty in order to overcome the hurdles posed by difficult anatomies.
